# Diagnostic accuracy of blood B-cell subset profiling and autoimmunity markers in Sjögren’s syndrome

**DOI:** 10.1186/ar4442

**Published:** 2014-01-17

**Authors:** Divi Cornec, Alain Saraux, Jacques-Olivier Pers, Sandrine Jousse-Joulin, Thierry Marhadour, Anne-Marie Roguedas-Contios, Steeve Genestet, Yves Renaudineau, Valérie Devauchelle-Pensec

**Affiliations:** 1 Service de Rhumatologie, Centre Hospitalier Régional et Universitaire de Brest, Hôpital de la Cavale Blanche, BP 824, F-29609 Brest cedex, France; 2 EA 2216 Immunologie et Pathologie, Université de Brest, SFR ScinBios, Labex Imunotherapy, Graft, Oncology, BP 824, F-29609 Brest cedex, France; 3 Service de Dermatologie, Centre Hospitalier Régional et Universitaire de Brest, Hôpital Morvan, BP 824, F-29609 Brest cedex, France; 4 Explorations Fonctionnelles Neurologiques, Centre Hospitalier Régional et Universitaire de Brest, Hôpital de la Cavale Blanche, BP 824, F-29609 Brest cedex, France

## Abstract

**Introduction:**

The aims of this study were to evaluate the diagnostic accuracy of blood B-cell subset profiling and immune-system activation marker assays in primary Sjögren’s syndrome (pSS) and to assess whether adding these tools to the current laboratory item would improve the American-European Consensus Group (AECG) criteria.

**Methods:**

In a single-center cohort of patients with suspected pSS, we tested the diagnostic performance of anti-SSA, antinuclear antibody (ANA), rheumatoid factor (RF), gammaglobulins, IgG titers, and B-cell ratio defined as (Bm2 + Bm2′)/(eBm5 + Bm5), determined using flow cytometry. The reference standard was a clinical diagnosis of pSS established by a panel of experts.

**Results:**

Of 181 patients included in the study, 77 had pSS. By logistic regression analysis, only ANA ≥1:640 (sensitivity, 70.4%; specificity 83.2%) and B-cell ratio ≥5 (sensitivity, 52.1%; specificity, 83.2%) showed independent associations with pSS of similar strength. In anti-SSA-negative patients, presence of either of these two criteria had 71.0% sensitivity but only 67.3% specificity for pSS; whereas combining both criteria had 96.2% specificity but only 12.9% sensitivity. Adding either of these two criteria to the AECG criteria set increased sensitivity from 83.1% to 90.9% but decreased specificity from 97.1% to 85.6%, whereas adding both criteria in combination did not substantially modify the diagnostic performance of the criteria set. The adjunction of RF + ANA ≥1:320, as proposed in the new American College of Rheumatology (ACR) criteria, did not improve the diagnostic value of anti-SSA.

**Conclusions:**

Blood B-cell subset profiling is a simple test that has good diagnostic properties for pSS. However, adding this test, with or without ANA positivity, does not improve current classification criteria.

## Introduction

Primary Sjögren’s syndrome (pSS) is a chronic autoimmune disorder that primarily affects the salivary and lachrymal glands. B cells play a major role in the pathogenesis of pSS [1]. Thus, biological markers for B-cell activity and autoimmunity might help to establish the diagnosis of pSS.

The main serological markers for pSS are autoantibodies against Ro/SSA or La/SSB ribonucleoproteins. These markers are the only biological item in the widely used American-European Consensus Group (AECG) classification criteria [2]. However, they are present in only 50% to 75% of patients with pSS [3] and are frequently encountered in other systemic autoimmune diseases [4]. Other biological markers may thus be valuable for the diagnosis of pSS.

Recently published American College of Rheumatology (ACR) classification criteria for pSS suggest that positivity for antinuclear antibodies (ANAs) and rheumatoid factor (RF) should be considered in patients negative for anti-Ro/SSA antibodies [5]. ANAs are present in 80% of patients with pSS and RF in 40% [3]. However, the sensitivity (Se) and specificity (Sp) of these tests for pSS compared to controls with other causes of sicca syndrome need to be assessed before accepting these as part of a criteria set.

B-cell activation may result in hypergammaglobulinemia, which is common in patients with pSS [3]. Immunoglobulin (Ig)A and IgG are often elevated and can display RF activity [6].

Sound evidence indicates that the distribution of peripheral-blood B-cell subsets is profoundly altered in patients with pSS. Memory B cells accumulate in target epithelial organs, and their proportion is decreased in peripheral blood [7]. On the other hand, the proportions of transitional and naive B cells are increased in peripheral blood [8]. IgD/CD38 staining was originally designed for B cell subset study in tonsils, separating important stages in B-cell development from naive to memory B cells (Bm1 to Bm5) [9]. This classification is helpful for studying blood B-cell subset alterations in pSS: in peripheral blood, Bm2 (IgD+/CD38 low) and Bm2′ (IgD+/CD38 high) populations include mainly transitional and activated naïve B cells and are increased in pSS patients, whereas eBm5 (IgD-/CD38 low) and Bm5 (IgD-/CD38-) populations are memory B cells, which are less represented in patients with pSS compared to patients with rheumatoid arthritis or normal control subjects [10]. We previously showed in a case–control study that these alterations in blood B-cell subset distribution may have an interesting diagnostic value for pSS. The better item to predict a diagnosis of pSS using only fluorescence-activated cell sorting (FACS) analysis was the B-cell ratio defined as (Bm2 + Bm2′)/(eBm5 + Bm5), which was strongly associated with pSS compared to rheumatoid arthritis, systemic lupus erythematosus, and healthy controls [11].

The primary aim of this study was to assess the diagnostic value of B-cell subset profiling and other biological autoimmunity markers in a cross-sectional cohort of patients with suspected pSS. We also evaluated the diagnostic usefulness of these tools compared to anti-SSA antibodies and other items of the AECG criteria set.

## Methods

### Study population

This prospective study was performed in a cohort of patients with suspected pSS recruited in Brittany, France, between November 2006 and September 2011 [12]. Inclusion criteria were subjective ocular or oral dryness, recurrent or bilateral parotidomegaly, or extraglandular symptoms suggestive of pSS. The study was approved by the local medical ethics committee (Brest University Hospital), and written informed consent was obtained from all patients before study inclusion.

### Clinical and laboratory evaluations

Each patient underwent a standardized assessment including a bilateral Schirmer’s test (abnormal if ≤5 mm/5 min on at least one side) and unstimulated salivary flow measurement (abnormal if <0.1 mL/min); a joint evaluation; and a general examination. Laboratory tests included serum protein electrophoresis; assays of IgG, IgA, and IgM; ANA on Hep2 cells, anti-SSA and anti-SSB antibodies using commercial ELISAs, and RF using in-house ELISA (IgM and IgA isotypes) [6]. Minor labial salivary gland biopsy (SGB) was graded according to the semi-quantitative Chisholm and Mason score [13].

### Blood B-cell subset profiling

**Figure 1:**
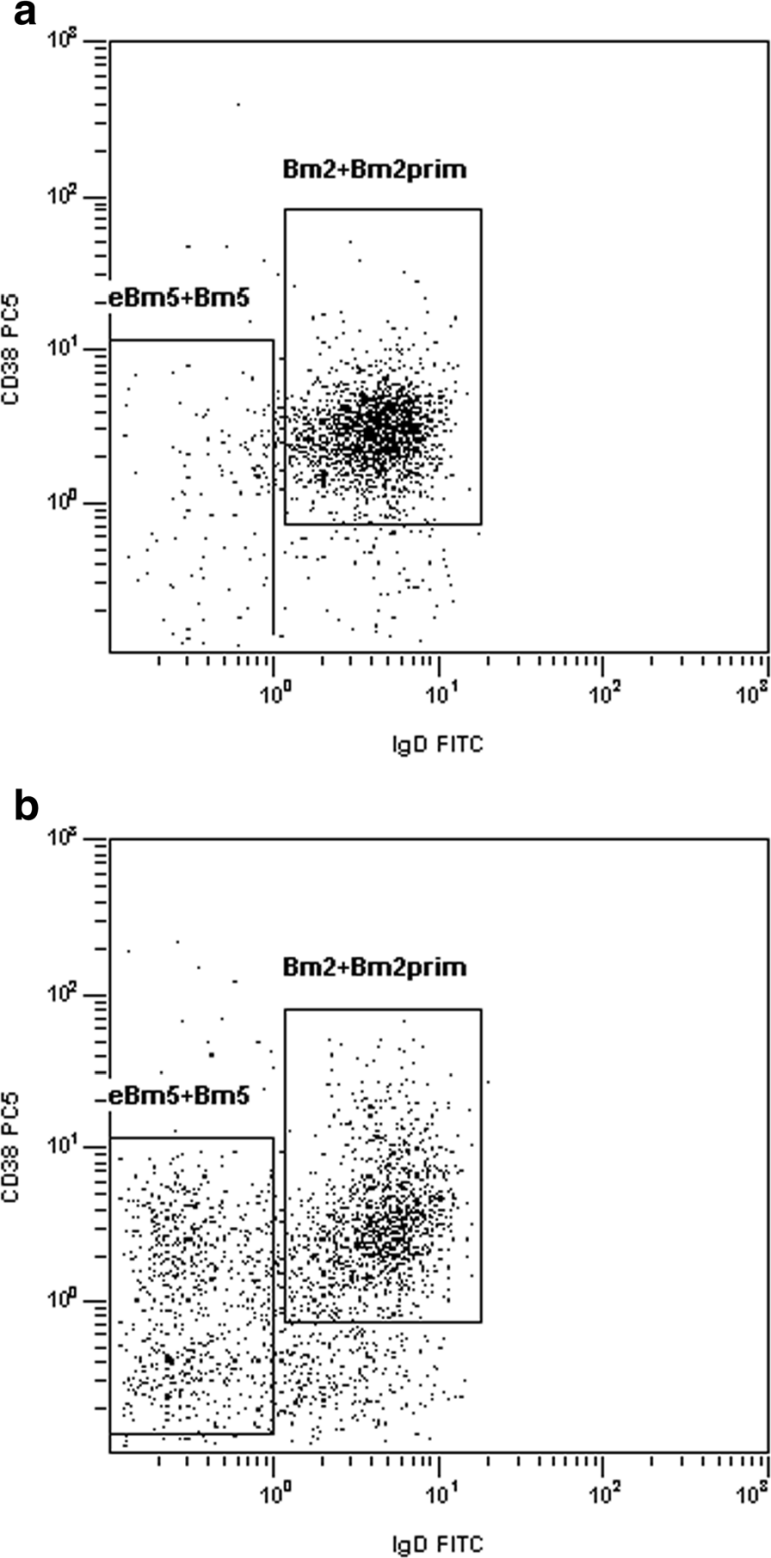
**Examples of typical blood B-cell subset profiling by flow cytometry.** All analyses are gated on CD19+ B cells. A ratio is computed between Bm2 + Bm2′ (immunoglobulin (Ig)D+/CD38*low-high* population) and eBm5 + Bm5 (IgD-/CD38*low-negative* population) subset proportions. **(a)** Primary Sjögren’s syndrome patient, with a ratio of 17.9. **(b)** Idiopathic sicca patient, with a ratio of 2.2.

Flow cytometry was performed as previously published [11]. The Bm2 + Bm2′ subset was defined as the IgD + and CD38*low/high* population, and the eBm5 + Bm5 subset as the IgD- and CD38*negative/low* population (Figure 1). The proportion of these two subsets in the total CD19+ B-cell population was determined, and the (Bm2 + Bm2′)/(eBm5 + Bm5) ratio (B-cell ratio) was computed.

### Reference standard

The reference standard was a clinical diagnosis of pSS performed by the evaluating rheumatologist, based on the clinical interview and examination, standard biology and salivary gland biopsy results. All doubtful cases were reviewed by a panel of three experts, who were blinded to the results of B-cell profiling. Other systemic diseases were diagnosed according to published classification criteria.

### Statistical analysis

Statistical tests were performed using the Statistical Package for the Social Sciences (SPSS 18.0, 2009, SPSS Inc., Chicago, IL, USA). Quantitative variables are described as means ± standard deviation and qualitative variables as numbers (%). We compared patients who fulfilled the reference standard (clinical diagnosis of pSS) to those who did not, using Mann–Whitney and chi-square tests as appropriate. We plotted receiver-operating characteristic (ROC) curves to determine the optimal cutoff associated with the best combination of sensitivity (Se) and specificity (Sp) for each test.

To determine which laboratory tests were independently associated with a diagnosis of pSS, we performed multiple logistic regression with backward selection using the likelihood ratio test. All items associated with a diagnosis of pSS by univariate analysis with *P* values <0.2 were included in this analysis.

## Results

Of the 181 patients included in the study (Table 1), 167 (92.2%) were women. Mean age was 56.1 ± 13.0 years and mean symptom duration was 6.4 ± 6.9 years. Of the 77 patients given a clinical diagnosis of pSS, 64 (83.1%) fulfilled AECG criteria. All AECG items were significantly associated with a diagnosis of pSS except xerophthalmia and xerostomia. No differences were found between pSS and non-pSS patients for age, disease duration, or sex ratio. Other diagnoses were: idiopathic sicca syndrome (N = 51), other systemic autoimmune diseases (N = 29), drug-induced sicca syndrome (N = 21), ill-controlled diabetes mellitus (N = 2), hepatitis C virus-related sicca syndrome (N = 1).

**Table 1:** Comparison of pSS and non-pSS patients

	**Overall**	**pSS**	**No pSS**	***P *** **value**
	**n = 181**	**n = 77**	**n = 104**	
Age (years, mean ± SD)	56.1 ± 13.0	56.3 ± 13.5	55.9 ± 12.7	0.89
Symptom duration (years, mean ± SD)	6.4 ± 6.9	7.0 ± 7.6	6.0 ± 6.3	0.48
Female, n (%)	167 (92.2)	70 (90.9)	97 (93.3)	0.56
Xerophthalmia, n (%)	156 (86.2)	70 (90.9)	86 (82.7)	0.11
Xerostomia, n (%)	166 (91.7)	73 (94.8)	93 (89.4)	0.19
Abnormal Schirmer’s test, n (%)	76 (42.0)	44 (57.1)	32 (30.8)	<0.001
Decreased salivary flow, n (%)	80 (44.2)	48 (62.3)	32 (30.8)	<0.001
Abnormal salivary gland biopsy, n (%)	75 (41.4)	62 (80.5)	13 (12.5)	<0.001
Anti-SSA or -SSB positivity, n (%)	47 (26.0)	47 (61.0)	0 (0.0)	<0.001
AECG criteria, n (%)	67 (37.0)	64 (83.1)	3 (2.9)	<0.001
Anti-SSA positivity, n (%)	47 (26.0)	47 (61.0)	0 (0.0)	<0.001
Anti-SSB positivity, n (%)	25 (13.8)	25 (32.5)	0 (0.0)	<0.001
ANA ≥1:320, n (%)	105 (58.0)	62 (80.5)	43 (41.3)	<0.001
ANA ≥1:640, n (%)	73 (40.3)	54 (70.1)	19 (18.3)	<0.001
IgM-RF positivity, n (%)	56 (30.9)	35 (45.5)	21 (20.2)	<0.001
IgA-RF positivity, n (%)	37 (20.4)	32 (41.6)	5 (4.8)	<0.001
Gammaglobulins ≥14 g/L, n (%)	44 (24.3)	36 (46.8)	8 (7.7)	<0.001
IgG ≥14 g/L, n (%)	47 (26.0)	38 (49.4)	9 (8.7)	<0.001
B-cell ratio ≥5, n (%)	59 (32.6)	41 (53.2)	18 (17.3)	<0.001
ANA ≥1:320 + IgM-RF, n (%)	39 (21.5)	32 (41.6)	7 (6.7)	<0.001
ACR criteria serologic item, n (%)	56 (30.9)	49 (63.6)	7 (6.7)	<0.001

pSS, primary Sjögren’s syndrome; AECG, American-European Consensus Group; ANA, antinuclear antibodies; Ig, immunoglobulin; RF, rheumatoid factor; ACR criteria serologic item: (anti-SSA/B or (ANA ≥1:320 + IgM-RF)). Xerophthalmia and xerostomia referred to subjective complaints by the patients. Schirmer’s test was considered abnormal if ≤5 mm/5 min and unstimulated salivary flow if ≤0.1 mL/min. Salivary gland biopsies were graded according to Chisholm and Mason. Mann–Whitney and chi-square tests were used as appropriate to compare pSS and non-pSS patients. *P* <0.05 was considered significant.

**Figure 2:**
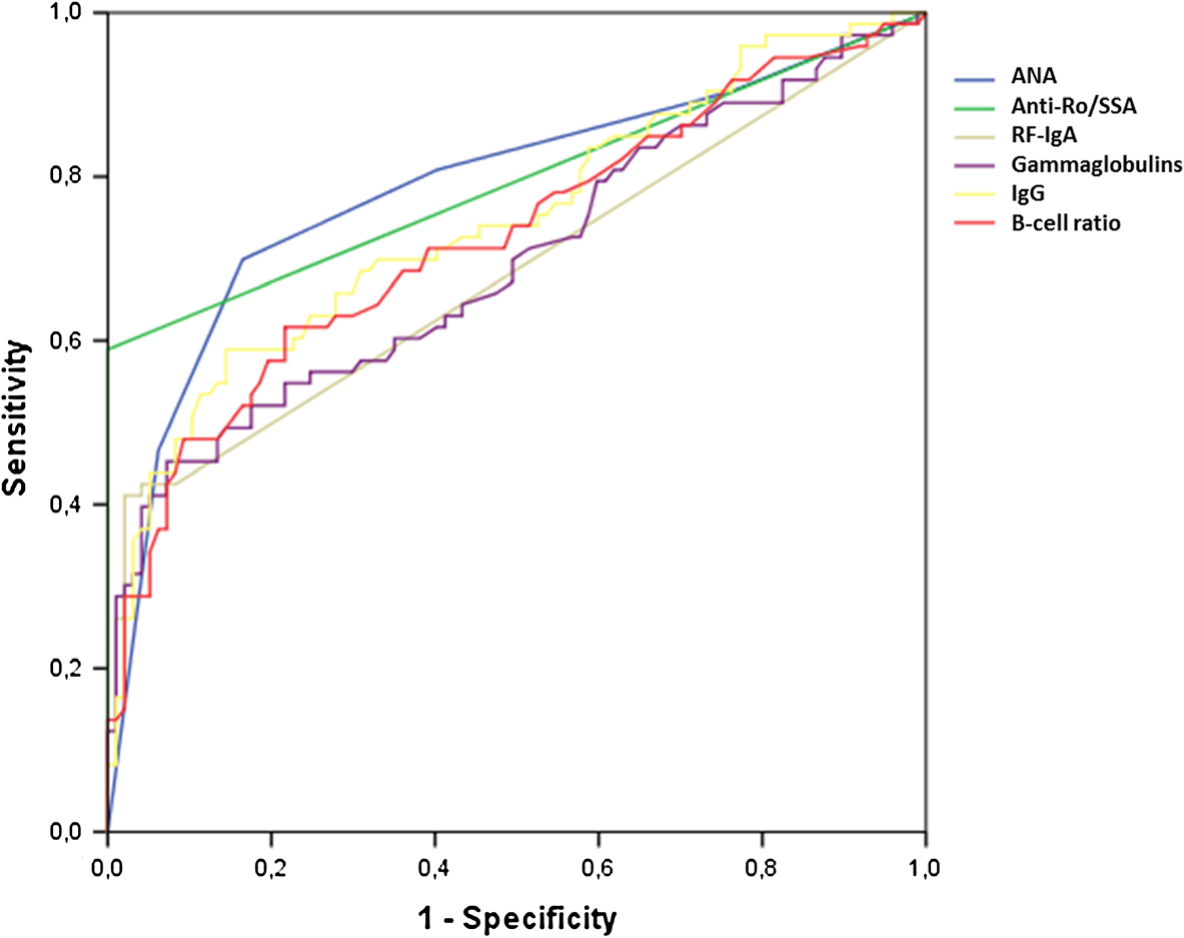
**ROC curve analysis.** The best cutoff for each test is chosen as the best combination of sensitivity and specificity. ROC, receiver-operating characteristic; ANA, antinuclear antibodies; RF, rheumatoid factor; Ig, immunoglobulin.

The mean B-cell ratio was significantly higher in the pSS group than in the non-pSS group (7.4 ± 6.9 vs. 3.2 ± 2.3, *P* <0.001). ROC curve analysis (Figure 2) identified ≥5 as the best cutoff, with 52.1% Se and 83.2% Sp. The highest values of this ratio were strongly suggestive of pSS, with a cutoff ≥6.5 having 92.6% Sp and a cutoff ≥9 97.9% Sp (with 42.3% and 28.2% Se, respectively). The correlation of B-cell ratio ≥5 with anti-SSA and abnormal SGB findings, estimated using Cohen’s kappa coefficient, was moderate (kappa = 0.35 and 0.30, respectively).

Figure 2 shows the ROC curve analysis for the biological markers. Their diagnostic value is shown in Table 2. Anti-SSB antibodies were found in 25 (32.5%) patients with pSS, all of whom also had anti-SSA antibodies. No patient had anti-SSA without ANA. ACR 2012 criteria serological item (anti-SSA/B or (ANA ≥1:320 + positive RF)) did not perform better than anti-SSA/B alone, but gave more false positive results.

**Table 2:** Diagnostic value of the biological items

	**Sensitivity**	**Specificity**	**PPV**	**NPV**
Anti-SSA or -SSB positivity	61.0%	100%	100%	79.9%
Anti-SSA positivity	61.0%	100%	100%	79.9%
Anti-SSB positivity	32.5%	100%	100%	66.7%
ANA ≥1:320	80.5%	58.7%	59.0%	80.3%
ANA ≥1:640	70.1%	81.7%	74.0%	78.7%
IgM-RF positivity	45.5%	79.8%	62.5%	66.4%
IgA-RF positivity	41.6%	95.2%	86.5%	68.8%
Gammaglobulins ≥14 g/L	46.8%	92.3%	81.8%	70.1%
IgG ≥14 g/L	49.4%	91.3%	80.9%	70.9%
B-cell ratio ≥5	53.2%	82.7%	69.5%	70.5%
ACR criteria serologic item	63.6%	93.7%	87.5%	77.6%

PPV, positive predictive value; NPV, negative predictive value; ANA, antinuclear antibodies; Ig, immunoglobulin; RF, rheumatoid factor; American College of Rheumatology (ACR) criteria serologic item: (anti-SSA/B or (ANA ≥1:320 + IgM-RF)).

Thirty patients were diagnosed as pSS but were anti-SSA negative. Among them, three patients had a normal SGB, and the diagnosis of pSS was made based on subjective and objective ocular and mouth dryness without other explanation, high-titer ANA, and suggestive extraglandular manifestations (cutaneous vasculitis, peripheral neuropathy, interstitial pneumonitis or cytopenia). In this group of anti-SSA negative pSS patients, the frequency of the different tests was: 56.7% for ANA ≥1:320; 40.0% for ANA ≥1:640; 20.0% for IgM-RF; 10.0% for ACR 2012 criteria serological item (anti-SSA/B or (ANA ≥1:320 + positive RF)); 10.0% for gammaglobulins ≥14 g/l; 16.7% for IgG ≥14 g/l; and 40.0% for B-cell ratio ≥5.

By logistic regression analysis, only anti-SSA antibodies, ANA ≥1:640 and B-cell ratio ≥5 showed independent associations with pSS. Using both ANA ≥1:640 and B-cell ratio ≥5 in combination had 37.7% Se and 96.2% Sp in the overall population but only 12.9% Se in the anti-SSA-negative subset. Using either ANA ≥1:640 or B-cell ratio ≥5 had 85.7% Se and 67.3% Sp in the overall group and 71.0% Se in the anti-SSA-negative subgroup.

Modifying the AECG criteria set by adding either ANA ≥1:640 or B-cell ratio ≥5 increased Se from 83.1% to 90.9%, but decreased Sp from 97.1% to 85.6%. Adding both ANA ≥1:640 and B-cell ratio ≥5 did not significantly modify the diagnostic performance of the criteria set (Se 84.2% vs. 83.1% and Sp 96.1% vs. 97.1%).

## Discussion

We assessed the diagnostic performance of autoimmunity and B-cell-related markers for pSS. Although ANA and RF positivity, hypergammaglobulinemia, IgG elevation, and altered peripheral-blood B-cell subset distribution were far more common in the patients with pSS compared to those with other causes of sicca symptoms, these abnormalities correlated closely with anti-SSA positivity. Only ANA ≥1:640 and B-cell ratio ≥5 were associated with pSS independently from anti-SSA antibodies. However, adding these two tests to the currently used AECG classification criteria did not improve their diagnostic performance.

Classification criteria are developed as research tools for establishing homogeneous patient groups with well-defined conditions and for comparing different studies, but they are widely used in clinical practice for diagnostic purposes. However, clinicians may diagnose a disease in patients who do not meet the classification criteria, especially those with recent-onset or mild symptoms. We studied a cohort of patients who reflected the diagnostic conditions encountered in everyday clinical practice, since they were referred to a specialized center for the evaluation of suspected pSS. The diagnosis was established by consensus among three experts independently from the classification criteria. This study design allowed us to assess the diagnostic performance of various tests and of the AECG criteria in the real-life setting. We previously used this methodology to study the diagnostic performance of salivary gland ultrasonography for pSS [12].

However, this methodology induces mandatorily a certain part of circular reasoning, since the experts in their own minds gave probably more weight for their diagnosis to known and objective features of pSS, such as anti-SSA antibodies or SGB biopsy results. If the experts would have taken into account the results of B-cell ratio (to which they were blinded) to make their diagnosis, they would probably have considered some patients as pSS due to a high B-cell ratio, which would have improved the diagnostic value of the test in this study.

Flow cytometry is simple, reproducible, and widely available. A high (Bm2 + Bm2′)/(eBm5 + Bm5) ratio is far more common in pSS both compared to other rheumatic diseases, as reported in our previous study [11], and compared to sicca syndromes not due to pSS, as shown here. Higher ratio values are associated with a higher probability of pSS. Thus, in the individual patient, this test is valuable. However, its diagnostic weight seems small compared to the other items of the AECG classification criteria set.

The abnormal distribution of blood B-cell subsets during pSS has previously been explored in regard to the disease pathophysiology. The decrease in blood memory B cells in pSS patients may be explained by their accumulation within salivary glands. Indeed, this variation partly reflects their migration into the exocrine glands of the patients [7], as well as into their skin [14]. We had previously observed that Bm2/Bm2′ cells express CD19 at 55% and 61% higher levels than the same cell subsets in normal controls [8]. Such marked increases could be related to pathogenesis, since modest increases in the density of CD19 are sufficient to shift the balance between tolerance and autoimmunity [15]. To address the functional significance of the observed phenotype anomalies, the dynamic translocation of protein into lipid rafts was also explored, since these domains are critical for proximal B cell receptor (BCR) signal transduction. The results revealed that the association of the BCR with the lipid rafts was prolonged in pSS. This may be accounted for by the overexpression of CD19, which prolongs signaling or prevents the recruitment of negative regulators, whether such regulators are insufficient (CD32) or altered (CD45) [8]. As such, changes in the lipid raft dynamics might lead to an aberrant B-cell response in pSS. Finally, these perturbations in B-cell homeostasis in pSS are closely correlated with the dysregulation of various cytokines regulating B-cell survival and activation, such as BAFF [8] and FLT3L [16].

ANA are frequent in patients with pSS. In the new preliminary ACR classification criteria set proposed by Shiboski *et al*. [5], the required ANA titer is 1:320, but this cutoff was chosen by consensus among experts and the article does not report a detailed Se and Sp analysis. In our study, the higher ANA titer of ≥1:640 was required to obtain the best combination of Se and Sp, and was associated with pSS independently from anti-SSA positivity. RF, and especially IgA-RF, are far more frequent in patients with pSS compared to controls. However, this test correlates strongly with anti-SSA positivity. Considering ANA titer and RF positivity beside anti-SSA for the diagnosis of pSS do not seem useful.

## Conclusions

This study shows that biological evidence of autoimmunity is common in patients with pSS but is closely related to the presence of anti-SSA antibodies. Anti-SSA remains the main serological tool for diagnosing pSS. Other serological markers should be tested in cohorts of patients with suspected pSS. The development of new classification criteria for pSS has been the focus of an international debate since the publication of the ACR 2012 criteria [17,18], and we believe that our cohort of patients with suspected pSS should be useful to validate such criteria.

## Abbreviations

ACR: American College of Rheumatology; AECG: American-European Consensus Group; ANA: antinuclear antibody; Ig: immunoglobulin; pSS: primary Sjögren’s syndrome; RF: rheumatoid factor; ROC: receiver-operating characteristic; Se: sensitivity; SGB: salivary gland biopsy; Sp: specificity.

## Competing interests

The authors have no competing interests to declare concerning this work.

## Authors’ contributions

DC, AS and VDP designed the study. DC, AS, JOP, SJJ, TM, AMRC, SG, YR and VDP participated in the management of the patients and their recruitment in the cohort, and in the acquisition of data. JOP and YR performed the biological assays. DC and AS performed the statistical analysis. DC and VDP drafted the manuscript. AS, JOP, SJJ, TM, AMRC, SG and YR critically revised the manuscript. All authors read and approved the final manuscript, and agree to be accountable for all aspects of the work.
